# Supplementary material for: Temperature sensitivity of DNA double-strand break repair underpins heat-induced meiotic failure in mouse spermatogenesis
Source: Commun Biol. 2022 May 26;5:504. doi: 10.1038/s42003-022-03449-y (PMC9135715; doi:10.1038/s42003-022-03449-y)
Supplement: Supplementary file 2 — Supplementary Information [file 42003_2022_3449_MOESM2_ESM.pdf]

Supplementary Information for

Temperature sensitivity of DNA double-strand break repair underpins heat-induced meiotic failure in mouse spermatogenesis

Kodai Hirano, Yuta Nonami, Yoshiaki Nakamura, Toshiyuki Sato, Takuya Sato, Kei-ichiro Ishiguro, Takehiko Ogawa, *Shosei Yoshida

Shosei Yoshida
Email: shosei@nibb.ac.jp

This PDF file includes:

Supplementary Figures 1 to 5
Supplementary Tables 1 to 3
Supplementary Refences

Supplementary Figure and Table

Supplementary Figure 1

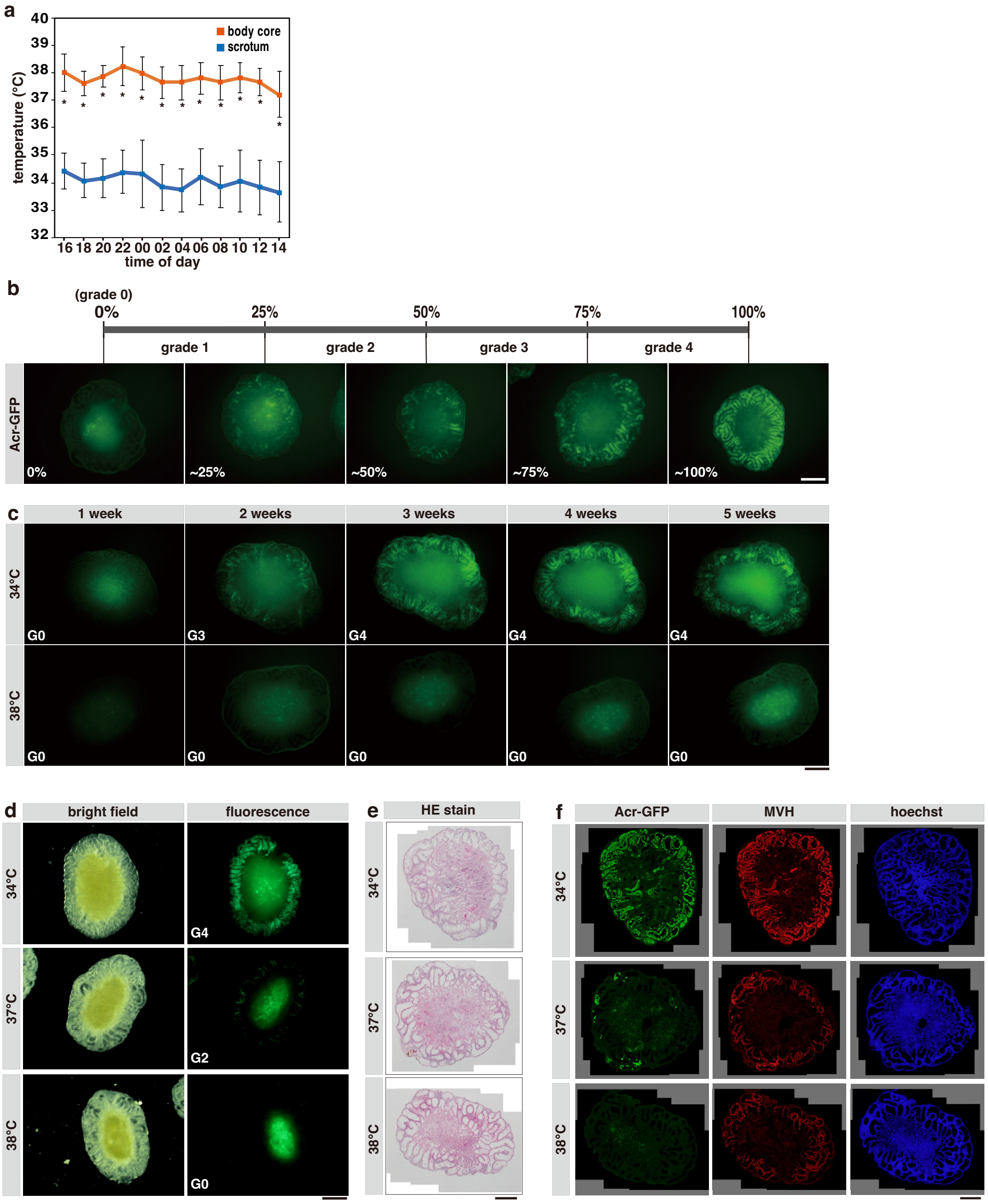

Supplementary Figure 1. Temperature condition and *ex vivo* explant culture of mouse testis, related to Figure 1

(a) Scrotal and abdominal temperatures in mice were measured using implanted thermometers at the indicated times of the day. The light and dark phases were at 8:00–20:00 and 20:00–8:00, respectively. All data points are shown as the mean ± SD (N = 14 individuals). *$p < 0.01$ between scrotal and abdominal temperatures. **(b)** Examples of fluorescence microscopy images of testicular explants in culture, indicating the grade of spermatogenesis progression based on *Acr-GFP* transgene expression. The extent of spermatogenesis was visually classified, based on the approximate percent area showing intense GFP signal in the peripheral region, into grades (G) 0 (no signal), 1 (0–25%), 2 (25–50%), 3 (50–75%), or 4 (75–100%). **(c)** Representative fluorescence images of the testis explants with the Acr-GFP transgene during five weeks of culture at 34 ℃ or 38 ℃, photographed once every week. GFP grade at each time point is indicated. Scale bar, 500 µm. **(d)** Representative bright-field (left) and fluorescence (right) microscopic images of the testis explant with the Acr-GFP transgene after 5weeks of culture at 34 ℃, 37 ℃, and 38 ℃, photographed after being transferred onto a microscope slide. GFP grade is indicated. Scale bar, 500 µm. **(e-f)** Whole images of sectioned *Acr-GFP* mouse testis explants after 5 weeks of culture at 34 ℃, 37 ℃, or 38 ℃, stained with hematoxylin-eosin (e), and for the indicated marker proteins (f). Scale bars: 500 µm.

Supplementary Figure 2

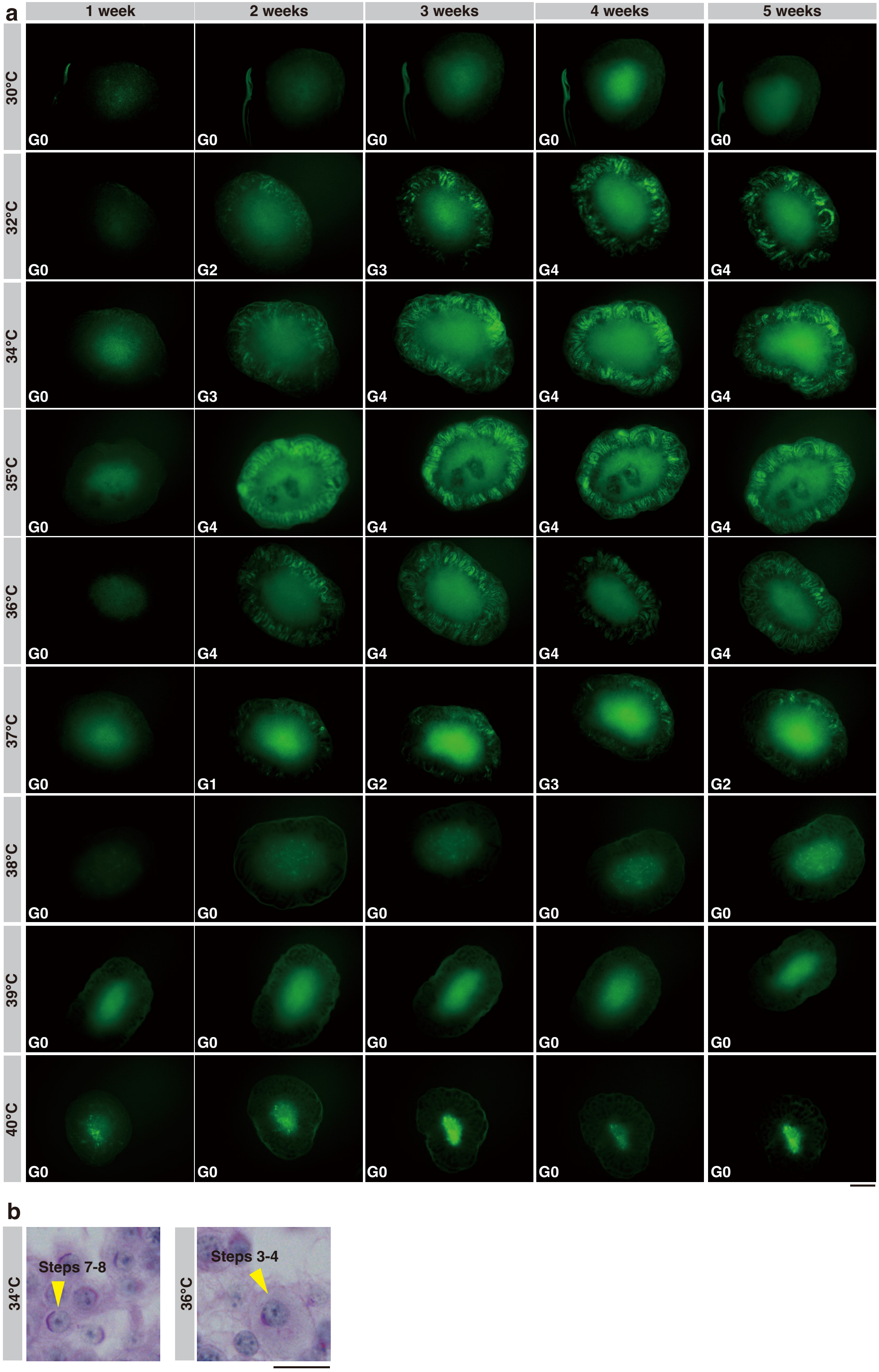

Supplementary Figure 2. Spermatogenesis progression in *ex vivo* testis explants at a range of different temperatures, related to Figure 2

(a) Fluorescence images of representative explants with Acr-GFP expression at the indicated temperatures and time points during five weeks of culture. Photographs of the same explants are also presented. Scale bar, 500 µm. Some photographs are also shown in Fig. 1e, 2a, and Supplementary Fig 1e. **(b)** Examples of round spermatids observed in PAS-hematoxylin-stained sections of testis explants cultured for five weeks at 34 ℃ and 36 ℃. Although advanced steps of round spermatids (e.g., steps 7–8 harboring large and curved acrosomes) were readily observed at 34 ℃, many round spermatids remained immature (e.g., up to steps 3–4 with smaller acrosomal bodies) at 36 ℃, as indicated by yellow arrows. Scale bar, 20 µm.

Supplementary Figure 3

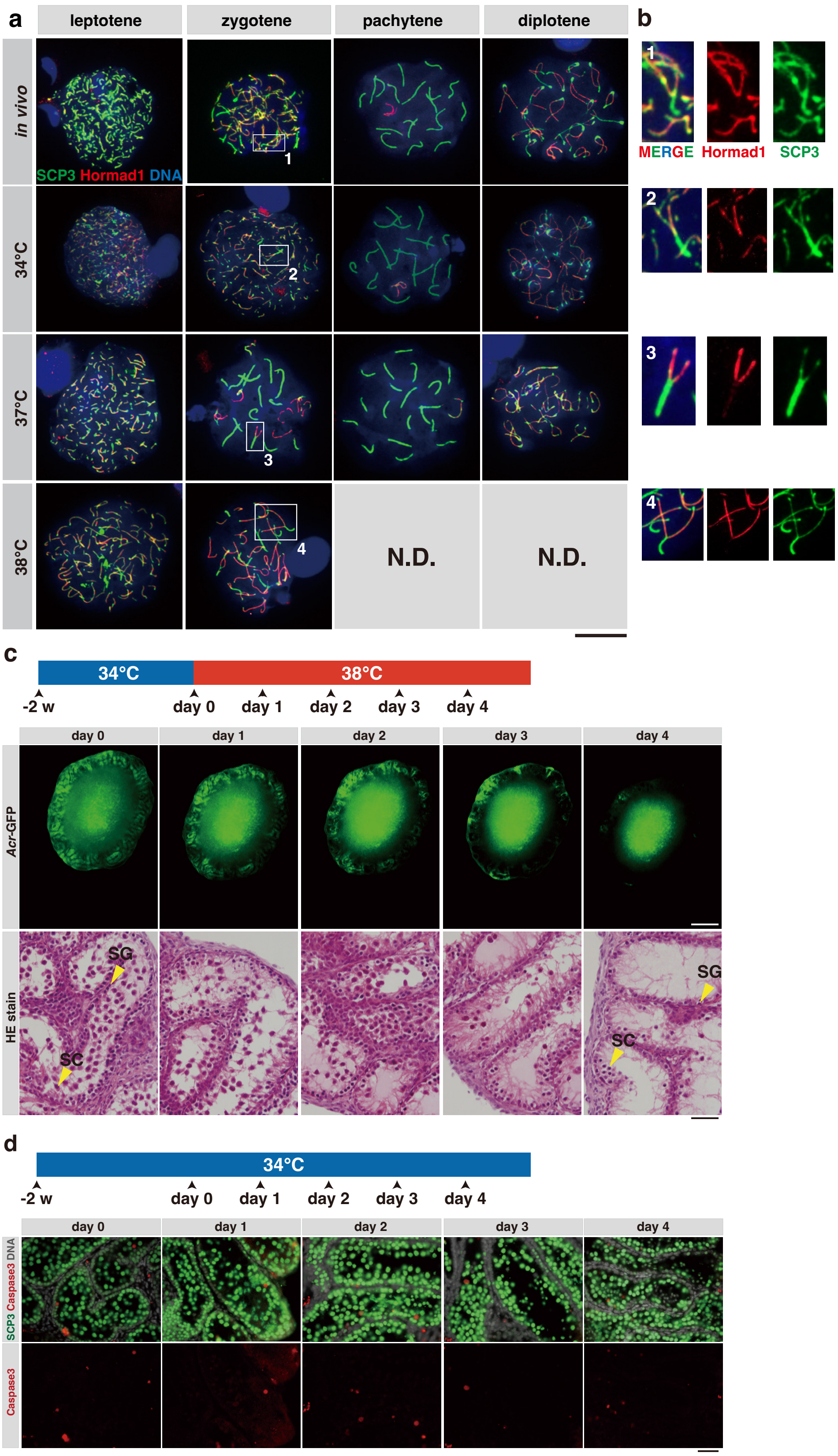

Supplementary Figure 3. Localization of chromosome axis proteins and impact of temperature shift in testis explants, related to Figure 3

(a-b) Representative images of chromosomal spreads at different stages of meiotic prophase I (i.e., leptotene, zygotene, pachytene), observed in the *in vivo* developed testes of 5-week-old *Acr-GFP* mice and explants cultured at 34 ℃, 37 ℃, or 38 ℃ for five weeks. Samples were stained for Hormad1 (red), SCP3 (green), and DNA (blue) in (a). Rectangles with numerals in (a) indicate the magnified area in (b). Scale bar, 20 µm. N.D., not detected. **(c)** Acr-GFP fluorescence (upper) and hematoxylin–eosin staining (lower) of testis explants cultured for 2 weeks at 34 ℃, followed by an additional 1–4 days at 38 ℃. Note the rapid disappearance of GFP signal and loss of spermatogenic cells following the temperature shift. SG, spermatogonia; SC, spermatocytes. **(d)** Detection of cleaved Caspase3 (an apoptosis marker, red) and SCP3 (green) without temperature shift. Double-staining images overlaid with DNA staining (gray) are shown in the upper panels; signals for cleaved Caspase3 are shown separately in the lower panels. Scale bars indicate 500 µm in the upper panels of (c) and 40 µm in the lower panels of (c) and (d), respectively.

Supplementary Figure 4

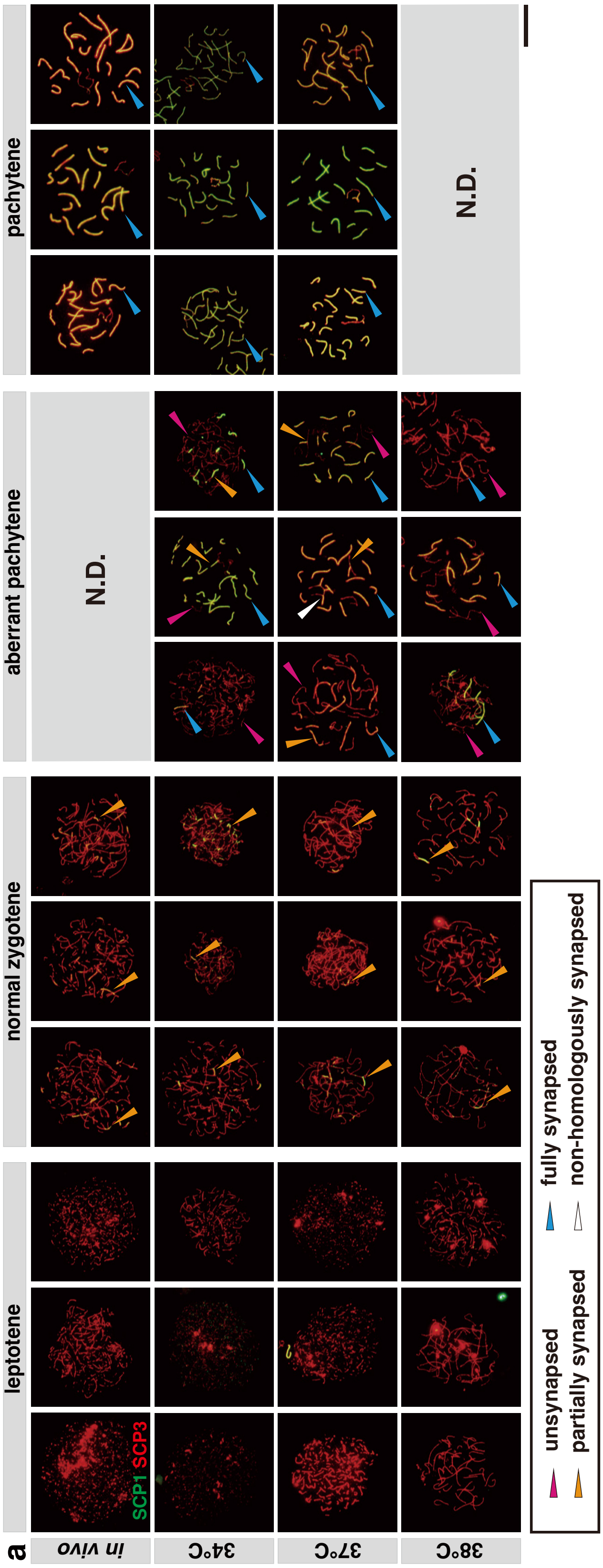

Supplementary Figure 4. Progression of chromosome pairing during meiotic prophase I at different temperatures, related to Figure 7

(a) Representative images of chromosomal spreads stained for SCP3 (red) and SCP1 (green), classified as leptotene, normal zygotene, aberrant pachytene, and (fully synapsed) pachytene spermatocytes, observed in *in vivo*-developed testes of 5-week-old *Acr-GFP* mice and explants cultured at 34 ℃, 37 ℃, or 38 ℃ for five weeks. Arrowheads indicate unsynapsed (red), partially synapsed (orange), fully synapsed (blue), or nonhomologous synapsed (white) chromosomes. Some photographs are shown in Fig. 7a. Scale bar, 20 µm. N.D., not detected.

Supplementary Figure 5

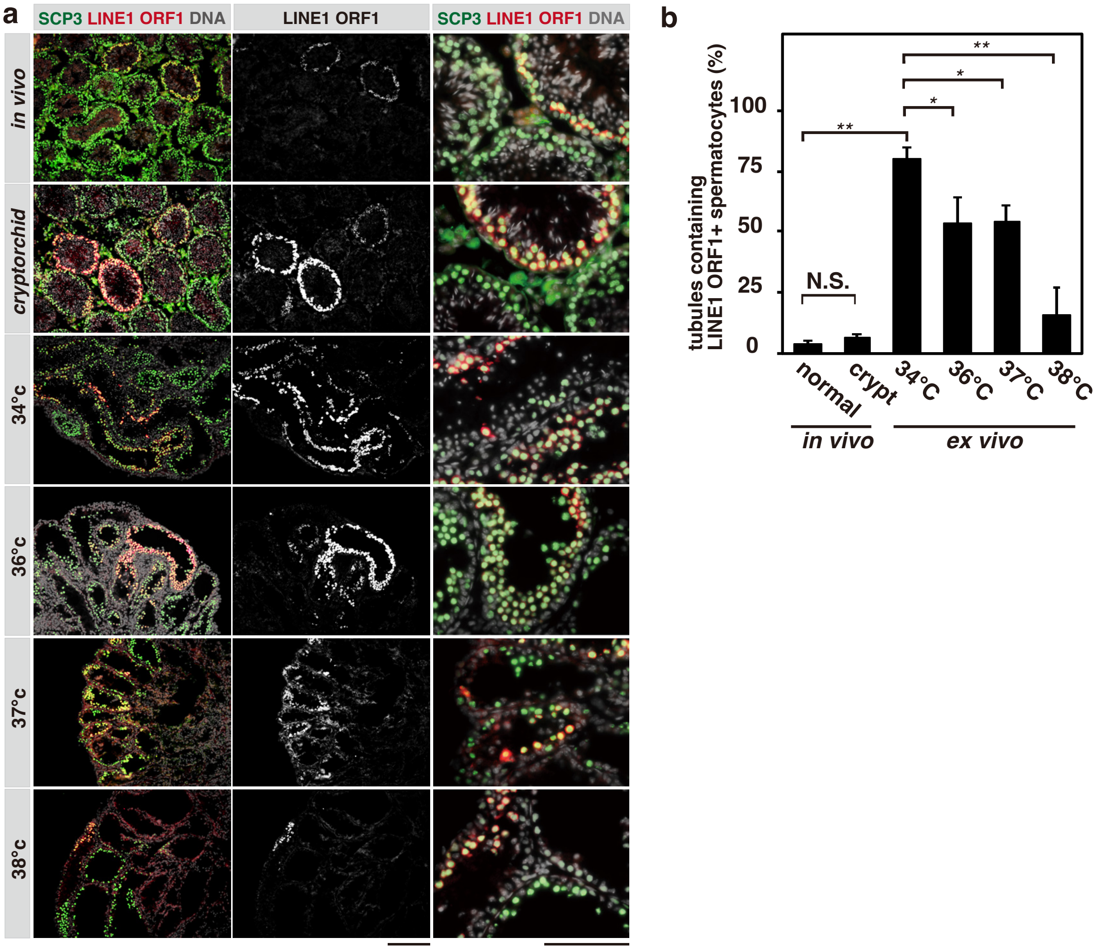

Supplementary Figure 5. Expression of LINE1 transposon in the testis at different temperatures *in vivo* and *ex vivo*

(a) Representative images of sections of normally developed testes of 5-week-old mice, artificial cryptorchid testes (two days after testis translocation), and testis explants cultured for five weeks at 34 ℃, 36 ℃, 37 ℃, and 38 ℃, stained for LINE1-ORF1 (red), SCP3 (green), and DNA (grey). Scale bars: 200 (left) and 100 µm (right). **(b)** Fractions of seminiferous tubules harboring LINE1 ORF1+/SCP3+ spermatocytes out of those with SCP3+ spermatocytes counted in testes developed *in vivo* and *ex vivo*, as shown in (a). Averaged percentage ± SEM values were calculated from five normally developed testes, five cryptorchid testes, and 5, 5, 6, and 4 explants cultured at 34 ℃, 36 ℃, 37 ℃, and 38 ℃, respectively. *$p < 0.05$, **$p < 0.01$ (t-test).

Supplementary Table 1.

Effect of temperature conditions on mouse spermatogenesis cultured ex vivo

Effect of temperature on mouse spermatogenesis

Temperature	
30°C	GFP expression was not observed over 5 weeks. The explants had few Mvh-positive cells in most seminiferous tubules (N = 3/7). A few spermatogonia and early spermatocytes were observed, but late spermatocytes as well as round and elongating spermatids were lost. Although a few GFRα1-, c-kit-, or SCP3-positive cells were noted, many seminiferous tubules had numerous GATA4-positive Sertoli cells in the peripheral area.
32°C	GFP expression was observed in the peripheral area of the explants from 2 to 5 weeks. The explants had many Mvh-positive cells in most seminiferous tubules. Spermatogonia, spermatocytes, and round spermatids were observed at 32°C, but the number of elongated spermatids decreased compared to that at 34°C (N = 4/4). GFRα1- or c-kit-positive cells as well as many SCP3-positive spermatocytes and GATA4-positive Sertoli cells were observed at the periphery of seminiferous tubules.
34°C	GFP expression was observed in the peripheral area of the explants from 2 to 5 weeks. The explants had many Mvh-positive cells in most seminiferous tubules. Spermatogonia, spermatocytes, as well as round and elongated spermatids were observed (N = 103/125). GFRα1- or c-kit-positive cells as well as many SCP3-spermatocytes and GATA4-positive Sertoli cells were observed at the periphery of seminiferous tubules.
35°C	GFP expression was observed in the peripheral area of the explants from 2 to 5 weeks. The explants had many Mvh-positive cells in most seminiferous tubules in the peripheral area. Spermatogonia, spermatocytes, and round spermatids were observed. Spermatogonia, spermatocytes, and round spermatids were observed at 35°C (N = 7/12), but the number of elongated spermatids decreased compared to that at 34°C. GFRα1- or c-kit-positive cells as well as many SCP3-spermatocytes and GATA4-positive Sertoli cells were observed at the periphery of seminiferous tubules.
36°C	GFP expression was observed in the peripheral area of the explants from 2 to 5 weeks. The explants had many Mvh-positive cells in most seminiferous tubules in the peripheral area. Spermatogonia, spermatocytes, and round spermatids were observed (N = 8/22). Unlike at 34°C, elongated spermatids were lost in all explants. The most advanced germ cell was step 3–4 spermatids (No. of explant = 3/4). GFRα1- or c-kit-positive cells as well as many SCP3-spermatocytes and GATA4-positive Sertoli cells were observed at the periphery of seminiferous tubules.
37°C	GFP expression was observed in half of the tubules in the peripheral area of the explants from 2 to 5 weeks. The explants had many Mvh-positive cells. Spermatogonia, early spermatocytes, and late spermatocytes were observed (N = 59/70). Unlike at 36°C, round and elongated spermatids were lost in all explants. The most advanced germ cell was late spermatocyte. GFRα1- or c-kit-positive cells as well as many SCP3-spermatocytes and GATA4-positive Sertoli cells were observed at the periphery of seminiferous tubules.
38°C	GFP expression was not observed over 5 weeks. The number of germ cells at the periphery of seminiferous tubules was lower than that observed at 34°C. Spermatogonia and early spermatocytes were observed (N = 33/33). Late spermatocytes as well as round and elongating spermatids were lost in all explants. The most advanced germ cell was zygotene spermatocyte (early spermatocyte). GFRα1- or c-kit-positive cells as well as many SCP3-spermatocytes and GATA4-positive Sertoli cells were observed at the periphery of seminiferous tubules.
39°C	GFP expression was not observed over 5 weeks. The explants had few Mvh-positive cells in most seminiferous tubules. A few spermatogonia and early spermatocytes were observed, but late spermatocytes as well as round and elongating spermatids were lost (N = 6/7). Although a few GFRα1-, c-kit-positive cells, or SCP3-positive cells were noted, many seminiferous tubules had many GATA4-positive Sertoli cells in the peripheral area.
40°C	GFP expression was not observed over 5 weeks. Although Mvh-positive cells were absent, many GATA4-positive Sertoli cells were observed in all explants (N = 13/13).

Supplementary Table 2. Antibodies used in this study

Antidoy (dillution)	Supplier	Catalog no and RRID
Goat polyclonal anti-GFRα1 (used at 1:1000)	R&D	Cat#AF560, RRID: AB_2110307
Rabbit polyclonal anti-SCP3 (used at 1:1000)	Abcam	Cat#ab15091, RRID: AB_301637
Mouse monoclonal anti-SCP3 (used at 1:1000)	Abcam	Cat#ab97672, RRID: AB_10678841
Rabbit polyclonal anti-SCP1 (used at 1:500)	NOVUS Biologicals	Cat#NB300-229, RRID: AB_10002742
Rabbit polyclonal anti-GATA4 (used at 1:500)	Thermo Fisher	Cat#PA1-102, RRID: AB_2539875
Rabbit monoclonal anti-MVH (used at 1:500)	Abcam	Cat#ab13840, RRID: AB_443012
Rabbit polyclonal anti-DMC1 (used at 1:1000)	Santa Cruz	Cat#sc-22768, RRID: AB_2277191
Goat polyclonal anti-c-Kit (used at 1:1000)	R&D	Cat#AF1356, RRID: AB_354750
Rabbit polyclonal anti-cleaved Caspase3 (Asp175) (used at 1:500)	Cell Signaling	Cat#9661, RRID: AB_2341188
Rat monoclonal anti-RPA32/RPA2 (4E4) (used at 1:80)	Cell Signaling	Cat#2208, RRID: AB_2238543
Rabbit polyclonal anti-HORMAD1 (used at 1:1000)	Proteintech	Cat# 13917-1-AP, RRID:AB_2120844
Mouse monoclonal anti-MLH1 (G168-15) (used at 1:100)	BD Biosciences	Cat# 551092, RRID:AB_394041
Rabbit polyclonal anti-RAD51 (used at 1:100)	Bioacademia	Cat#70-002
Rabbit polyclonal anti-phospho-H2A.X (Ser139; used at 1:500)	Millipore	Cat#07-164, RRID: AB_11213838
Rabbit polyclonal anti-LINE1 ORF1 (used at 1:5000)	Donal O'Carroll, The University of Edinburgh, UK	(Di Giacomo, et al., 2014)[1]
Donkey polyclonal anti-goat IgG (Alexa Fluor 594) (used at 1:1000)	Thermo Fisher	Cat#A11058, RRID: AB_2534105
Donkey polyclonal anti-goat IgG (Alexa Fluor 488) (used at 1:1000)	Jackson ImmunoResearch	Cat#705-545-147 RRID: AB_2336933
Donkey polyclonal anti-goat IgG (Alexa Fluor 594) (used at 1:1000)	Jackson ImmunoResearch	Cat#705-586-147, RRID: AB_2340434
Donkey polyclonal anti-mouse IgG (Alexa Fluor 488) (used at 1:1000)	Thermo Fisher	Cat#A21202, RRID: AB_141607
Donkey polyclonal anti-mouse IgG (Alexa Fluor 594) (used at 1:1000)	Jackson ImmunoResearch	Cat#715-585-151, RRID: AB_2340855
Donkey polyclonal anti-rabbit IgG (Alexa Fluor 488) (used at 1:1000)	Thermo Fisher	Cat#A21206, RRID: AB_2535792
Donkey polyclonal anti-rabbit IgG (Alexa Fluor 594) (used at 1:1000)	Thermo Fisher	Cat#A21207, RRID: AB_141637
Donkey polyclonal anti-rabbit IgG (Alexa Fluor 488) (used at 1:1000)	Jackson ImmunoResearch	Cat#711-545-152, RRID: AB_2313584

Supplementary Table 3. **Software used in this study**

Software	Supplier	URL
cellSens	Olympus	https://www.olympus-lifescience.com/en/software/cellsens/image-processing-and-sharing/
Photoshop (21.1.0)	Adobe	https://www.adobe.com/jp/products/photoshop.html
Excel 2019	Microsoft	https://www.microsoft.com/ja-jp/microsoft-365/excel
Illustrator (24.0.3)	Adobe	https://www.adobe.com/jp/products/illustrator.html
Kaleida Graph (4.5.0)	HULINKS	https://www.hulinks.co.jp/software/stat_graph/kaleida
R (4.0.5)	The R Foundation	http://www.R-project.org
BellCurve for Excel (2.00)	Social Survey Research Information Co., Ltd.	https://bellcurve.jp/ex/
Fiji (2.1.0/1.53c)	Wayne Rasband (NIH)	http://imagej.net
LAS X (3.5.5.19976)	Leica microsystems	https://www.leica-microsystems.com/

Supplementary References

1. Di Giacomo, M., Comazzetto, S., Sampath, S.C., Sampath, S.C. & O'Carroll, D. G9a co-suppresses LINE1 elements in spermatogonia. *Epigenetics Chromatin* **7**, 24 (2014).
