# Supplementary material for: Temperature sensitivity of DNA double-strand break repair underpins heat-induced meiotic failure in mouse spermatogenesis
Source: Commun Biol. 2022 May 26;5:504. doi: 10.1038/s42003-022-03449-y (PMC9135715; doi:10.1038/s42003-022-03449-y)
Supplement: Supplementary file 3 — Description of Additional Supplementary Files [file 42003_2022_3449_MOESM3_ESM.pdf]

Description of Additional Supplementary Files

File name: Supplementary Data 1
Description: Source data behind the graphs.
