# Supplementary material for: Temperature sensitivity of DNA double-strand break repair underpins heat-induced meiotic failure in mouse spermatogenesis
Source: Commun Biol. 2022 May 26;5:504. doi: 10.1038/s42003-022-03449-y (PMC9135715; doi:10.1038/s42003-022-03449-y)
Supplement: Supplementary file 5 — Reporting Summary [file 42003_2022_3449_MOESM5_ESM.pdf]

nature portfolio

Corresponding author(s): Shosei Yoshida

Last updated by author(s): Apr 2, 2022

Reporting Summary

Nature Portfolio wishes to improve the reproducibility of the work that we publish. This form provides structure for consistency and transparency in reporting. For further information on Nature Portfolio policies, see our Editorial Policies and the Editorial Policy Checklist.

Statistics

For all statistical analyses, confirm that the following items are present in the figure legend, table legend, main text, or Methods section.

n/a	Confirmed	
☐	☒	The exact sample size (*n*) for each experimental group/condition, given as a discrete number and unit of measurement
☐	☒	A statement on whether measurements were taken from distinct samples or whether the same sample was measured repeatedly
☐	☒	The statistical test(s) used AND whether they are one- or two-sided<br>*Only common tests should be described solely by name; describe more complex techniques in the Methods section.*
☒	☐	A description of all covariates tested
☒	☐	A description of any assumptions or corrections, such as tests of normality and adjustment for multiple comparisons
☐	☒	A full description of the statistical parameters including central tendency (e.g. means) or other basic estimates (e.g. regression coefficient) AND variation (e.g. standard deviation) or associated estimates of uncertainty (e.g. confidence intervals)
☐	☒	For null hypothesis testing, the test statistic (e.g. *F*, *t*, *r*) with confidence intervals, effect sizes, degrees of freedom and *P* value noted<br>*Give P values as exact values whenever suitable.*
☒	☐	For Bayesian analysis, information on the choice of priors and Markov chain Monte Carlo settings
☒	☐	For hierarchical and complex designs, identification of the appropriate level for tests and full reporting of outcomes
☒	☐	Estimates of effect sizes (e.g. Cohen's *d*, Pearson's *r*), indicating how they were calculated

Our web collection on statistics for biologists contains articles on many of the points above.

Software and code

Policy information about availability of computer code

Data collection	Microscopy:<br>- CellSense Standard 1.18<br>- LAS X 3.5.5.19976
Data analysis	Imaging:<br>- CellSense Standard 1.18<br>- Photoshop 21.1.0<br><br>Statistical analysis:<br>- Microsoft Excel for Mac 16.40<br>- Kaleida Graph 4.5.0<br>- R 4.0.5<br>- BellCurve for Excel 2.00<br>- Fiji 2.1.0

For manuscripts utilizing custom algorithms or software that are central to the research but not yet described in published literature, software must be made available to editors and reviewers. We strongly encourage code deposition in a community repository (e.g. GitHub). See the Nature Portfolio guidelines for submitting code & software for further information.

Data

Policy information about availability of data

All manuscripts must include a data availability statement. This statement should provide the following information, where applicable:

- Accession codes, unique identifiers, or web links for publicly available datasets
- A description of any restrictions on data availability
- For clinical datasets or third party data, please ensure that the statement adheres to our policy

All data supporting the finding of this study are available from the corresponding author on reasonable request.

Field-specific reporting

Please select the one below that is the best fit for your research. If you are not sure, read the appropriate sections before making your selection.

☒ Life sciences ☐ Behavioural & social sciences ☐ Ecological, evolutionary & environmental sciences

For a reference copy of the document with all sections, see nature.com/documents/nr-reporting-summary-flat.pdf

Life sciences study design

All studies must disclose on these points even when the disclosure is negative.

Sample size	No statistical methods were used to predetermine sample size.
Data exclusions	N/A
Replication	Technical or biological replicates are indicated in figure legends and methods. All replicates successfully reproduced the presented findings unless particularly stated. Otherwise, the number of replicates showing the presented findings are indicated.
Randomization	Animals were distributed at random over experimental groups.
Blinding	Investigators were not blinded to allocation during experiments.

Reporting for specific materials, systems and methods

We require information from authors about some types of materials, experimental systems and methods used in many studies. Here, indicate whether each material, system or method listed is relevant to your study. If you are not sure if a list item applies to your research, read the appropriate section before selecting a response.

Materials & experimental systems

n/a	Involved in the study	
☐	☒	Antibodies
☒	☐	Eukaryotic cell lines
☒	☐	Palaeontology and archaeology
☐	☒	Animals and other organisms
☒	☐	Human research participants
☒	☐	Clinical data
☒	☐	Dual use research of concern

Methods

n/a	Involved in the study	
☒	☐	ChIP-seq
☒	☐	Flow cytometry
☒	☐	MRI-based neuroimaging

Antibodies

Antibodies used

The following antibodies were used for immunostaining: goat polyclonal anti-GFRα1 (0.5ug/ml, R&D, AF560, RRID: AB_2110307), mouse monoclonal anti-SCP3 (1:1000, Abcam, ab97672, RRID: AB_10678841), mouse polyclonal anti-SCP3 (1:1000, Abcam, ab15091, RRID: AB_3011637), rabbit polyclonal anti-SCP1 (1:500, NOVUS Biologicals, NB300-229, RRID: AB_10002742), rabbit polyclonal anti-GATA4 (1:500, Thermo Fisher, PA1-102, RRID: AB_2539875), rabbit monoclonal anti-MVH (1:500, Abcam, ab13840, RRID: AB_443012), rabbit polyclonal anti-DMC1 (1:1000, Santa Cruz, sc-22768, RRID: AB_2277191), goat polyclonal anti-c-kit (1:1000, R&D, AF1356, RRID: AB_354750), rabbit polyclonal anti-cleaved Caspase3 (Asp175) (1:500, Cell Signaling, 9661, RRID: AB_2341188), rabbit polyclonal anti-phospho-H2A.X (1:500, Millipore, 07-164, RRID: AB_11213838), rabbit polyclonal anti-LINE1 orf1 (1:5000, Donal O'Carroll, The University of Edinburgh, Di Giacomo, et al., 2014). anti-RPA2 (Cell Signaling Technologies, #2208, 1:80, RRID: AB_2238543); anti-RAD51(Bio Academia, #70-002, 1:100); anti-MLH1 (BD Biosciences, #551092, 1:100, RRID:AB_394041); anti-HORMAD1 (proteintech, #13917-1-AP, 1:1000, RRID:AB_2120844). Secondary antibodies were Alexa Fluor-conjugated (488 or 594) from Thermo Fisher or Jackson ImmunoResearch and used at 1:1000 dilutions. The following secondary antibodies were used for immunostaing: Donkey polyclonal anti-goat IgG (Alexa Fluor 594) (1:1000, Thermo Fisher, A11058, RRID: AB_2534105), Donkey

polyclonal anti-goat IgG (Alexa Fluor 488) (1:1000, Jackson ImmunoResearch, 705-545-147, RRID: AB_2336933), Donkey polyclonal anti-goat IgG (Alexa Fluor 594) (1:1000, Jackson ImmunoResearch, 705-586-147, RRID: AB_2340434), Donkey polyclonal anti-mouse IgG (Alexa Fluor 488) (1:1000, Thermo Fisher, A21202, RRID: AB_141607), Donkey polyclonal anti-mouse IgG (Alexa Fluor 594) (1:1000, Jackson ImmunoResearch, 715-585-151, RRID: AB_2340855), Donkey polyclonal anti-rabbit IgG (Alexa Fluor 488) (1:1000, Thermo Fisher, A21206, RRID: AB_253792), Donkey polyclonal anti-rabbit IgG (Alexa Fluor 594) (1:1000, Thermo Fisher, A21207, RRID: AB_141637), Donkey polyclonal anti-rabbit IgG (Alexa Fluor 488) (1:1000, Jackson ImmunoResearch, 711-545-152, RRID: AB_2313584).

Validation

Goat polyclonal anti-GFRα1 (0.5ug/ml, R&D, AF560, RRID: AB_2110307) was validated in Nakagawa et al., Science 2010. Mouse monoclonal anti-SCP3 (1:1000, Abcam, ab97672, RRID: AB_10678841) was validated by the manufacturer (https://www.abcam.co.jp/scp3-antibody-cor-10g117-ab97672.html). Rabbit polyclonal anti-SCP3 (1:1000, Abcam, ab15091, RRID: AB_3011637) was validated by the manufacture (https://www.abcam.co.jp/scp3-antibody-ab15093.html). Rabbit polyclonal anti-SCP1 (1:500, NOVUS Biologicals, NB300-229, RRID: AB_10002742) was validated by the manufacture (https://www.novusbio.com/products/scp1-antibody_nb300-229#protocols-faqs). Rabbit polyclonal anti-GATA4 (1:500, Thermo Fisher, PA1-102, RRID: AB_2539875) was validated by the manufacture (https://www.thermofisher.com/antibody/product/GATA4-Antibody-Polyclonal/PA1-102). Rabbit monoclonal anti-MVH (1:500, Abcam, ab13840, RRID: AB_443012) was validated by the manufacture (https://www.abcam.co.jp/ddx4--mvh-antibody-ab13840.html). Rabbit polyclonal anti-DMC1 (1:1000, Santa Cruz, sc-22768, RRID: AB_2277191) was validated by the manufacture (https://www.scbt.com/ja/p/dmc1-antibody-h-100). Goat polyclonal anti-c-kit (1:1000, R&D, AF1356, RRID: AB_354750) was validated by the manufacture (https://www.rndsystems.com/products/human-mouse-cd117-c-kit-antibody_af1356#product-details). Rabbit polyclonal anti-cleaved Caspase3 (Asp175) (1:500, Cell Signaling, 9661, RRID: AB_2341188) was validated by the manufacture (https://www.cellsignal.jp/products/primary-antibodies/cleaved-caspase-3-asp175-antibody/9661). Rabbit polyclonal anti-phospho-H2A.X (1:500, Millipore, 07-164, RRID: AB_11213838) was validated by the manufacture (https://www.merckmillipore.com/JP/ja/product/Anti-phospho-H2A.X-Ser139-Antibody,MM_NF-07-164). Rabbit polyclonal anti-LINE1 orf1 was validated in Di Giacomo, et al., 2014. Rat monoclonal anti-RPA2 (Cell Signaling Technologies, #2208, 1:80, RRID: AB_2238543) was validated by the manufacture (https://www.cellsignal.com/products/primary-antibodies/rpa32-rpa2-4e4-rat-mab/2208). Rabbit polyclonal anti-RAD51(Bio Academia, #70-002, 1:100) was validated by the manufacture (http://www.bioacademia.co.jp/en/html/upload/save_image/E70-001%20anti-Rad51(human)antibody(rabbit-serum).pdf). Mouse monoclonal anti-MLH1 (BD Biosciences, #551092, 1:100, RRID:AB_394041) was validated by the manufacture (https://www.bdbiosciences.com/ja-jp/products/reagents/microscopy-imaging-reagents/immunohistochemistry-reagents/purified-mouse-anti-mlh-1-with-control.551092). Rabbit polyclonal anti-HORMAD1 (proteintech, #13917-1-AP, 1:1000, RRID:AB_2120844) was validated by the manufacture (https://www.ptglab.com/products/HORMAD1-Antibody-13917-1-AP.htm).
Donkey polyclonal anti-goat IgG (Alexa Fluor 594) (1:1000, Thermo Fisher, A11058, RRID: AB_2534105) was validated by the manufacture (https://www.thermofisher.com/antibody/product/Donkey-anti-Goat-IgG-H-L-Cross-Adsorbed-Secondary-Antibody-Polyclonal/A-11058). Donkey polyclonal anti-goat IgG (Alexa Fluor 488) (1:1000, Jackson ImmunoResearch, 705-545-147, RRID: AB_2336933) was validated by the manufacture (https://www.jacksonimmuno.com/catalog/products/705-545-147). Donkey polyclonal anti-goat IgG (Alexa Fluor 594) (1:1000, Jackson ImmunoResearch, 705-586-147, RRID: AB_2340434) was validated by the manufacture (https://www.jacksonimmuno.com/catalog/products/705-586-147). Donkey polyclonal anti-mouse IgG (Alexa Fluor 488) (1:1000, Thermo Fisher, A21202, RRID: AB_141607) was validated by the manufacture (https://www.thermofisher.com/antibody/product/Donkey-anti-Mouse-IgG-H-L-Highly-Cross-Adsorbed-Secondary-Antibody-Polyclonal/A-21202).
Donkey polyclonal anti-mouse IgG (Alexa Fluor 594) (1:1000, Jackson ImmunoResearch, 715-585-151, RRID: AB_2340855) was validated by the manufacture (https://www.jacksonimmuno.com/catalog/products/715-585-151). Donkey polyclonal anti-rabbit IgG (Alexa Fluor 488) (1:1000, Thermo Fisher, A21206, RRID: AB_253792) was validated by the manufacture (https://www.thermofisher.com/antibody/product/Donkey-anti-Rabbit-IgG-H-L-Highly-Cross-Adsorbed-Secondary-Antibody-Polyclonal/A-21206). Donkey polyclonal anti-rabbit IgG (Alexa Fluor 594) (1:1000, Thermo Fisher, A21207, RRID: AB_141637) was validated by the manufacture (https://www.thermofisher.com/antibody/product/Donkey-anti-Rabbit-IgG-H-L-Highly-Cross-Adsorbed-Secondary-Antibody-Polyclonal/A-21207). Donkey polyclonal anti-rabbit IgG (Alexa Fluor 488) (1:1000, Jackson ImmunoResearch, 711-545-152, RRID: AB_2313584) was validated by the manufacture (https://www.jacksonimmuno.com/catalog/products/711-545-152).

Animals and other organisms

Policy information about studies involving animals; ARRIVE guidelines recommended for reporting animal research

Laboratory animals

Acr-GFP transgenic mice (B6;B6C3-Tg(Acro3-EGFP)01Osb (RIKEN BRC, #RBRC00886)) were crossed to C57BL/6J (Japan SLC and Japan CLEA). 4-day-old and 5-8-week-old male Acr-GFP taransgenic mice and 8-week-old male C57BL/6J mice were used.

Wild animals

This study does not involve wild animals.

Field-collected samples

This study does not involve samples collected from the field.

Ethics oversight

All mouse experiments were conducted with the approval of the Institutional Animal Care and Use Committee of the National Institutes of National Sciences.

Note that full information on the approval of the study protocol must also be provided in the manuscript.
